# Spectrum of maternal and perinatal outcomes among parturient women with preceding short inter-pregnancy interval at Bugando Medical Centre, Tanzania

**DOI:** 10.1186/s40748-014-0002-1

**Published:** 2015-01-22

**Authors:** Athanase Lilungulu, Dismas Matovelo, Albert Kihunrwa, Balthazar Gumodoka

**Affiliations:** Department of Obstetrics & Gynecology, Catholic University of Health & Allied sciences, P.O. BOX 1464, Mwanza, Tanzania; Department of Obstetrics & Gynecology, Bugando Medical Centre, P.O. BOX 1370, Mwanza, Tanzania

**Keywords:** Short inter-pregnancy interval, Normal inter-pregnancy interval, Maternal and perinatal outcome

## Abstract

**Background:**

Traditionally women with a short inter-pregnancy interval will not have sufficient time to recover and get ready for the subsequent pregnancy. This includes socio-economic, cultural, psychological and physical body preparedness. The present study aimed at comparing the maternal and perinatal outcomes among parturient women with preceding short and normal inter-pregnancy interval attending at Bugando Medical Centre (BMC). This was a prospective cohort study. It was done from November 2012 to April 2013. Multiple matching design approach was used to adjust for age variable during selection of participants. Chi-square test and Relative Risk (RR) were calculated to test for strength of association between variables.

**Results:**

Four hundred and fifty (450) women were recruited in this study in which 150 had a SIPI and 300 had a NIPI. The premature rupture of membrane (PROM) was higher [RR = 13.6; 95% CI 7.2 − 25.6] among SIPI women than in NIPI women [RR = 0.57; 95% CI 0.49–0.7]. Women with a SIPI were found to have a significantly higher risk for anemia (RR = 3.4) compared to those with a NIPI (RR = 0.08). SIPI women had a higher risk for failure of trial of vaginal birth after caesarean section (VBAC) (RR = 14.7; 95% CI 6.4 − 33.6) compared to NIPI (RR = 0.72; 95% CI 0.65–0.8). The risk of postpartum hemorrhage (PPH) was higher among SIPI women (RR = 5.8) compared to women of NIPI (RR = 0.83). Women with SIPI had higher risk for small for gestation age (SGA) babies (RR = 7.7; 95% CI 3.8-15.7), low birth weight (RR = 6.7; 95% CI 3.6-12.3), preterm delivery (RR = 9.78; 95% CI 4.9-19.5) and low Apgar score (RR = 6.9; 95% CI 0.7-0.8) compared to women in NIPI.

**Conclusion:**

Higher risk for PROM, anemia, failure of trial of VBAC, PPH and preeclampsia were observed among women with SIPI. Babies born of mothers with a SIPI were significantly at higher risk for SGA, low birth weight, low Apgar score, preterm deliveries compared to women in NIPI.

Birth spacing, creating more awareness of complications, on risks associated with SIPI and provision of folate supplements should be advocated.

## Background

Birth interval is an important determinant of the rates of population growth and socio-economic status of communities. It offers a great potential in protecting the health status of the mothers, and improving outcome of subsequent pregnancy [1].

A short inter-pregnancy interval (SIPI) is a period between delivery of the previous infant and conception of the current pregnancy of less than 18 months [2]. This remains to be a major challenge among women in developing countries associated with increased risk for maternal and neonatal mortality [2,3]. Normal inter-pregnancy interval (NIPI) is the period between delivery of the previous infant and conception of the current pregnancy of 18 to 36 months. The solution for SIPI can only be achieved through use of different contraception methods [4]. Despite the understanding and promotion of women’s care; maternal and fetal adverse outcomes are reported to be high among SIPI women [5].

The impact of SIPI is greater in very young women; this is because an immature adolescent who is still growing, may compete with the fetus for nutrients [6]. Pregnant women with short interval have increased risk of uterine rupture or scar dehiscence, failure trial of scar, placenta abruption, placenta previa, antenatal and perinatal infections [7]. Studies have shown higher burden of maternal and child mortality in Tanzania and other developing countries and there is limited information regarding the effect of SIPI on maternal and fetal adverse outcome [8]. Maternal mortality in Tanzania is 454 deaths per 100000 live births with Neonatal mortality of 26 deaths per 1000 live births [9].

The maternal and fetal outcomes among parturient women with SIPI compared with NIPI in our setting have not been determined. Determination of obstetric outcomes among women with SIPI will help fine-tune efforts towards accelerating attainment of MDG5 [10].

## Results

### Study population characteristics

The study was conducted between November 2012 and April 2013 at BMC labour ward. During the study period 450 pregnant women participated in this study in which 150 were exposed characterized by SIPI and 300 pregnant with NIPI as unexposed group. Women were recruited in matched group’s study design done by same age between 20 to 25 years old or ±1 year on the same day of admission.

The mean age of the participants was 23.4 ± 1.7. Majority of participants 446(99.1%) had only primary school education. Secondary school education in both SIPI and NIPI groups 3 (0.7%) and only 1 (0.2%) participant both for SIPI and NIPI had highest education level respectively, among 450 participants 449 (99.8%) were married. There was no statistically significant difference between the two groups with regard to socio-demographic variables (Table 1).

**Table 1:** **Social demographic characteristics of SIPI & NIPI women**

**Variable**	**Overall value**	**Study groups**	
		**SIPI (n = 150)**	**NIPI (n = 300)**
**Mean women** Age ± S.D.*	23.4 ± 1.7	23.3 ± 1.6	23.4 ± 1.7
**Education status**			
Primary	446 (99.1)	148 (98.7)	298 (99.3)
Secondary/Higher Education	4 (0.9)	2 (1.3)	2 (0.7)
**Occupation**			
Housewife	447 (99.3)	149 (99.3)	298 (99.3)
Self-employment	3 (0.7)	1 (0.7)	2 (0.7)
**Marital status**			
Married	449 (99.8)	149 (99.3)	300 (100)
Single	1 (0.2)	1 (0.7)	-
**Parity**			
Two	268 (59.5)	86 (57.3)	182 (60.7)
Three	138 (30.7)	49 (32.7)	89 (29.7)
More than Four	44 (9.8)	15 (10)	29 (9.6)

^*^S.D = Standard deviation.

### Obstetric outcomes in SIPI and NIPI women

The majority of the SIPI were anemic 141 (94.0%) as compared to only 83 (27.7%) of the NIPI. The observed difference was statistically significant p < 0.01. Forty four (29.3%) of the SIPI had failure of trial of scar as compared to 6 (2.0%) of the NIPI, the observed difference was statistically significant p < 0.01. Again, 29 (19.3%) of SIPI had postpartum haemorrhage as compared to 10 (3.3%) of the NIPI the observed difference was statistically significant. There were more women in the SIPI group that had premature rupture of membranes 68 (45.3%) as than to those in the NIPI group which were only 10 (3.3%) and the observed difference was significant p < 0.01. Preeclampsia was seen in 27 (18.0%) of SIPI as compared to 8 (2.7%) of NIPI the observed difference was statistically significant p < 0.01 (Table 2).

**Table 2:** **Maternal adverse outcome among women with SIPI and NIPI**

	**Inter-pregnant interval**		
	**SIPI N = 150**	**NIPI N = 300**	
	**n(%)**	**n(%)**	**p-value**
**Hemoglobin concentration**			
<11 g/dl	141(94.0)	83(27.7)	**<**0.01
≥11 g/dl	9(6.0)	217(72.3)	
**Trial of VBAC**			**˝**
Failed	44(29.3)	6(2.0)	
Successful	106(70.7)	294(98.0)	
Pre eclampsia*			**˝**
Present	27(18.0)	8(2.7)	
Absent	123(82.0)	292(97.3)	
**Postpartum hemorrhage**			**˝**
Present	29(19.3)	10(3.3)	
Absent	121(80.7)	290(96.7)	
**PROM**			**˝**
Present	68(45.3)	10(3.3)	
Absent	82(54.7)	290(96.7)	

*Preeclampsia was defined as an increase in blood pressure to at least 140/90 mm Hg after the 20th week of gestation, an increase in diastolic blood pressure of at least 15 mm Hg or an increase in systolic blood pressure of at least 30 mm Hg from the level measured before the 20th week, combined with proteinuria (protein excretion, at least 0.3 g per 24 hours).

### Perinatal outcomes in SIPI and NIPI women

There were more babies born with low birth weight among women in SIPI group 40 (26.7%) as compared to only 12 (4.0%) of the NIPI, the observed difference was statistically significant p < 0.01. Prematurity was seen in 44 (29.3%) of the SIPI as compared to 9 (3.0%) of the NIPI, the observed difference was statistically significant p < 0.01. There were more babies who are small for gestation age (SGA) from women in the SIPI group 35 (26.7%) as compared to 9 (3.0%) of the NIPI, the observed difference was statistically significant p < 0.01. Most of the SIPI babies had low score 38 (25.6%) as compared to only 11 (3.7%) of the NIPI; the observed difference was statistically significant p < 0.01. Most of the babies of SIPI women had prolonged hospital stay 123 (82%) as compared to 27 (9.0) babies of the NIPI women, the observed difference was statistically significant p < 0.01 (Table 3).

**Table 3:** **Perinatal adverse outcome among women with SIPI and NIPI**

	**Inter-pregnancy interval**		
	**SIPI N = 150**	**NIPI N = 300**	
	**n(%)**	**n(%)**	**p-value**
**Birth weight**			
<2500 g	40(26.7)	12(4.0)	**<**0.01
≥2500 g	110(77.3)	288(96.0)	
**Prematurity**			
Present	44(29.3)	9(3.0)	**˝**
Absent	106(70.7)	291(97.0)	
**Length of hosp stay**			
>24 hrs.	123(82)	27(9.0)	**˝**
≤24 hrs.	27(18)	273(91)	
**SGA**			
Present	35(23.3)	9(3.0)	**˝**
Absent	115(76.7)	291(97.0)	
**Apgar score at 5th minute**			
0-6	38(25.6)	11(3.7)	**˝**
7-10	112(74.7)	289(96.3)	

### The relative risk of maternal and fetal adverse outcome among SIPI and NIPI women

Women with a SIPI had higher risk for PROM compared to those with NIPI [RR, 13.6; 95% CI 7.2 − 25.64]. Women with a SIPI were found to have a significantly higher risk for anemia [RR = 3.4; 95% CI 2.8 − 4.1] compared to NIPI women. Failure of VBAC was higher among women with SIPI as compared to NIPI [RR = 14.7; 95% CI 6.4 − 33.6]. The risk of preeclampsia was higher among SIPI as compared to NIPI [RR = 6.8; 95% CI 3.1 − 14.5]. SIPI women had a higher risk for postpartum haemorrhage as compared to those with NIPI [RR = 5.8; 95% CI 2.9 − 11.6]. Regarding fetal adverse outcomes, it was found that women in SIPI had delivered preterm babies compared to NIPI women [RR = 9.8; 95% CI 4.9-19.5]. The low Apgar score babies were higher among SIPI women compared to NIPI women [RR = 6.9; 95% CI 3.6 − 13.1]. There were more babies born with low birth weight among women in the SIPI as compared to NIPI [RR = 6.7; 95% CI 3.6 − 12.3]. Women with SIPI had higher SGA babies as compared to women in NIPI [RR = 7.7; 3.8–15.7] (Table 4).

**Table 4:** **Relative risk of maternal and fetal adverse outcome among women with SIPI and NIPI**

**Variable**	**Relative risk**	**95% CI**	***p-*** **value**
**Maternal adverse Outcome**			
PROM	13.6	7.2 – 25.64	<0.01
Anemia status	3.4	2.8 – 4.1	**˝**
Pre-eclampsia	6.75	3.14 –14.49	**˝**
PPH	5.8	2.9 – 11.58	**˝**
Failure of trial of VBAC	14.67	6.39 – 33.64	**˝**
**Fetal adverse Outcome**			
Pre-maturity	9.78	4.9 – 19.5	<0.01
Low score	6.9	3.6 – 13.1	**˝**
LBW	6.7	3.6 – 12.3	**˝**
SGA	7.7	3.8 – 15.74	**˝**

## Discussion

This study has demonstrated a very clear higher risk for maternal and neonatal adverse outcomes among women with SIPI than those with NIPI. This is because SIPI women will not have sufficient time to recover in terms of socio-economic, cultural, psychological and physical body preparedness and get ready for the subsequent pregnancy.

The study showed that failure of vaginal birth after caesarian section (VBAC) was 29.3% in SIPI than in NIPI women. This finding is similar to results from studies done in Denmark and Florida-USA where SIPI women had a significantly greater risk of having a failure trial of VBAC than in NIPI women [11,12]. The reason for failed VBAC was due to the poor progress of labour and early meconium stained liquor during the course of labour monitoring. In our study there was association between SIPI and failure of trial of VBAC, the clinical aspect of this reason may not be clearly seen as there is a need for another study to evaluating the rate of failure of VBAC for SIPI women attempting VBAC. However, SIPI may cause inadequate time for the postpartum healing of the previous caesarean section scar.

The study showed that postpartum hemorrhage was 19.3% in SIPI than in NIPI women. This is similar to other studies which also revealed an increased risk for PPH in SIPI than in NIPI women [13-15]. The PPH occurs due to interference in the endometrial blood vessels remodeling after delivery. It has been observed that SIPI is also the risk factor for PPH since, there are inadequate space interval for reproductive organs to have adequate resting period to carry for another pregnancy physiological changes [16,17]. The complications related to SIPI can be averted by longer and optimal spacing.

Anemia was prominently seen among women with a SIPI; the finding was similar to study done in Nigeria [18]. Women with SIPI were more likely to have anemia during the course of their pregnancy than NIPI women. Reason for anemia in SIPI women is seen to be due to shorter time to replenish the iron stores leading to total iron depletion [16].

The study showed increased risk for PROM women, thus putting them at risk for intrauterine infections. The finding is similar to that found in a systemic review done in 2012 by Conde agunelo et al. which revealed that PROM was 60% in SIPI women than in NIPI [19]. SIPI women with subclinical genital infections may continue to carry the organisms for several weeks to months after delivery leading to increased risks for PROM and later chorioamnionitis.

Pre eclampsia was 18.0% in SIPI women in this study. This finding was similar from the systemic review done in 2006 by Conde Agudelo et al. reported that SIPI women of less than 6 or 13 months were associated with increased risk of preeclampsia. The reason for this occurrence of preeclampsia was due to short birth space of one year, thus putting women at risk of having preeclampsia [20,21]. Our study has limitations in which we do not have data on smoking, obesity and change of partners which might have confounding effect on the association between SIPI and risk of preeclampsia.

In this study it was found that infants of birth weight below 2500 g were 26.7% in SIPI than in NIPI. This finding was found in other studies done in Qatar, England and Tanzania [22-25]. Low birth weight in SIPI women may be as the results of poor maternal nutrition due to short birth spacing and poor maternal weight gain [26,27]. This can be secondary to iron storage capacity and folate depletion in the body which increases women susceptibility to anemia and compromised nutritional status.

The study showed that preterm delivery was 29.3% in SIPI than NIPI women. The finding is similar to other studies which revealed an increased incidence of prematurity in SIPI women [2,28]. The reason for SIPI related prematurity may be related to high prevalence of anemia in this study and PROM causing chorioamnionitis. Histological chorioamnionitis by itself appears to be an independent risk factor for prematurity [16]. To overcome such burden of increasing preterm delivery, there is a need for a well-organized perinatal intensive care unit which will be able to provide all necessary health care services in preterm babies.

The study revealed that SGA was 23.3% in SIPI than in NIPI women. The finding was similar to other studies that showed SIPI women have high tendencies to deliver small for gestation age babies. According to the study done at Israel Zedek Medical Centre (IZMC), there was increased risk of SGA (8.5% vs. 7.6%) [29]. Similarly another study done in Netherlands found that SIPI women had high risk of delivering infants with SGA (22.8%) vs. (11.5%). The hypothetical explanation for SGA in SIPI women is due to poor maternal nutritional status and pregnancy folate storage capacity depletion syndrome from the previous pregnancy that is commonly seen in SIPI pregnant women [30]. Folic acid has a major role in DNA synthesis and cell division leading to an increased demand for folate during pregnancy. Thus, lowered maternal folate concentrations may negatively impair fetal growth and development.

This study showed that babies with low Apgar score were 25.6% in SIPI than in NIPI women. This indicates that majority of babies needed postnatal observations and evaluations after delivery. Study done in Morogoro Tanzania showed that there is increasing chance of low Apgar score when there was inter-pregnancy interval of less than 12 months [24]. The reason for low Apgar score in SIPI women may be related to low birth weight and prematurity which are commonly seen in SIPI women. Premature and low birth weight babies are prone to fetal distress during labor and hence low Apgar score. In this study; two babies were found to have congenital anomalies which are not commonly predicted for women with SIPI in case studies. However, none was found in NIPI, but this was not statistically significant [31]. The possible anticipation for congenital malformation could be Neural Tube Defects (NTD) which is mainly due to folate depletion in women with SIPI. Folate is highly needed during embryogenesis as it is time for tissue growth and sustained cell division.

The study also revealed that babies with prolonged hospital stay were 82.0% in SIPI than in NIPI women. Similar finding of increased higher risk for antenatal and postnatal morbidity for women with SIPI were seen in systematic review and study done in Cairo [19,32]. This shows that babies in SIPI women will need special attention post-delivery in terms of observation, assessment, care, investigations and management during the entire hospital stay hence more cost to their family and health facilities. The prolonged hospital stay may also cause impaired psychological of the mother with regard to their acceptability of the situation as well as poor perinatal surveillance prediction.

## Conclusion

Women with a SIPI are at a higher risk for anemia in pregnancy, PROM, preeclampsia and failure of VBAC, PPH than those with a NIPI. Also, babies born from SIPI women are at higher risk for low birth weight, low Apgar score, preterm delivery and small for gestation age than those with a NIPI. Couples and community at large should be advised on importance of child spacing and understand the major risk or complications of SIPI.

### Limitation of the study

We relied on mother’s recall for previous child date of birth and her last normal menstrual period. During the entire period of follow up by mobile phone it was difficult to have exactly information from the clients because of the unavailability of mobile phone network systems. This might have led to underestimation of the Relative Risk (RR) of the adverse outcomes.

## Methods

### Study design

The study was a prospective cohort study conducted among women giving birth at Bugando Medical Centre (BMC) over six months period from November 2012 to April 2013. Matching design of selection technique was used to adjust for age of the women during the entire period of study. The centre is one of the four referral hospitals in the country and it is located in Mwanza city in the northwestern part of Tanzania. On average 600 deliveries take place at BMC every month and BMC provide maternity services to low as well as high risk pregnant women referred in from peripheral facilities.

### Study population

Pregnant women with SIPI and NIPI upon the time of admission consented to participate in the study at BMC labor ward. All pregnant women in SIPI and NIPI groups attending at BMC labor ward during study period and voluntarily consented to participate in the study were included. Women with medical conditions in pregnancy and complicated previous deliveries were excluded from the study.

### Sampling technique

All eligible women in labour with SIPI and NIPI were selected purposively basing on their age between 20 and 25 years old. These women were categorized into exposed (SIPI) against unexposed (NIPI) groups of the same age until the desired number of exposed was reached, the methods of adjusting plus or minus one year from women of unexposed group was used and the ratio of exposed versus matched unexposed were either 1: 2 or adjusted by ±1 year.

### Data collection procedure

Prior to matching; the antenatal care (ANC) card was reviewed for either the last normal menstrual period (LNMP) or presence of an early ultrasonography done at ANC booking and date of the previous delivery. All women with SIPI and NIPI who met the inclusion criteria and gave consent to participate were recruited into the study. Once the eligibility has been established; explanation of the study objectives, consent form assigned and a case record of information were collected by using a questionnaire. Information on socio-demographic characteristics; obstetric history such as gravidity, parity and gestational age; mode of delivery, maternal and neonatal adverse outcomes including birth weight, Apgar score, SGA and admission to NICU were collected. The matched design was done upon the time of admission at the labour ward. A woman who was in labour and identified to be eligible for the study was recruited.

### Follow up

Each pregnant woman who was recruited in the study was followed up for obstetric outcomes and detailed information on the mother and the baby was recorded. Fetal outcomes including Apgar scores at the 5th minute, requirement for neonatal resuscitation, admission in neonatal intensive care unit, premature unit or a perinatal death. Birth asphyxia i.e., low Apgar score was diagnosed when baby did not take spontaneous respiration at birth and Apgar scores of less than seven after 5 minutes from birth. All babies were followed up to 7th day after delivery. All babies with adverse outcome were either admitted in neonatal intensive care unit (NICU) or premature/postnatal units. Babies without adverse outcome were given to their mothers for breast feeding; observed for 24 hours and later followed up by mobile phone for 7 days. Mothers with adverse outcome were admitted and managed based on hospital protocol and those without adverse outcome were discharged home after 24 hours, followed up by mobile phone for 7 days. For women without mobile phones; they were followed up at the postnatal clinic visit on the 7th day post-delivery. Two babies born with abnormalities and intrauterine fetal death were recorded, but were not included in the study as such two additional women with SIPI were enrolled to meet the required sample size.

### Data analysis

Data was analyzed using SPSS software version 17.0 (SPSS, Chicago IL, U.S.A). Results were summarized in the form of proportions for categorical variables. Means, standard deviations at 95% confidence level or median were used to summarize continuous variables. Chi-square test used to test for significance of associations between the predictor and outcome variables in the categorical variables. Relative risk was calculated to test for strength of association between variables. A p-value of less than 0.05 at 95% Confidence interval was considered significant.

### Ethical approval

Ethical approval to conduct the study was obtained from the joint Catholic University of Health & Allied Health Sciences and Bugando medical centre research ethics committee. Patient’s refusal to consent or withdraw from the study did not alter or jeopardize their access to medical care.

### Consent

All participants were provided with consent information sheet and forms to read and consent for participation, but for non literate women, the consent sheet was read aloud in Kiswahili by the recruiter. After agreeing to participate, her thumbprint was stamped on the consent form to signify her consent.
